# Can galvanic skin conductance be used as an objective indicator of children’s anxiety in the dental setting?

**DOI:** 10.4317/jced.53419

**Published:** 2017-03-01

**Authors:** Ebrahim Najafpour, Naser Asl-Aminabadi, Sara Nuroloyuni, Zahra Jamali, Sajjad Shirazi

**Affiliations:** 1 Assistant Professor, Department of Pediatric Dentistry, Faculty of Dentistry, Tabriz University of Medical Sciences, Tabriz, Iran; 2 Professor, Department of Pediatric Dentistry, Faculty of Dentistry, Tabriz University of Medical Sciences, Tabriz, Iran; 3 Assistant Professor, Department of Pediatric Dentistry, Ardabil University of Medical Sciences, Ardabil, Iran; 4 Associate Professor, Department of Oral Sciences, Faculty of Dentistry, Tabriz University of Medical Sciences, Tabriz, Iran; 5 Research Fellow and Lecturer, Dental and Periodental Research Centre, Faculty of Dentistry, Tabriz University of Medical Sciences, Tabriz, Iran; 6 Biotechnology Research Centre, Tabriz University of Medical Sciences, Tabriz, Iran

## Abstract

**Background:**

Assessment of procedural distress is essential at assisting children during invasive dental treatments. This study aimed to determine the validity and reliability of galvanic skin response as a measure for assessment of dental anxiety in children.

**Material and Methods:**

151 children, aged 5-7 years, participated in this study. Similar dental treatments were rendered to all subjects. At the beginning and end of the session, modified child dental anxiety scale (MCDAS), clinical anxiety rating scale (CARS) and galvanic skin response (GSR) were used to determine children’s anxiety.

**Results:**

GSR was significantly correlated with both MCDAS (rs=0.62, *p*=0.02) and CARS (rs=0.44, *p*=0.032). The correlation between MCDAS and CARS was also significant (rs = 0.9, *P*<0.001). Anxiety decreased during the session in both GSR (rs=0.52, *p*=0.001) and MCDAS scales (rs=0.77, *p*=0.001). CARS also showed a reduction between the initial and second assessment, but it was not statistically significant (rs=0.12, *P*=0.36).

**Conclusions:**

The findings suggest that GSR is a reliable and valid measure for assessment of children’s dental anxiety in the clinical context. GSR may help to identify clinically anxious children before dental treatment to provide appropriate interventions.

** Key words:**Dental anxiety, reliability, validity, galvanic skin response.

## Introduction

Despite the ever growing body of medical research, there is a paucity of relevant and comprehensive measurement tools for pediatric distress. It is important that clinically anxious children are identified as early as possible and are provided with appropriate interventions. Since different treatment strategies require different diagnostic categories, it is imperative that practitioners not only identify anxious patients, but also determine the severity of their anxiety. While relaxation and desensitization are effective with the simple conditioned phobias, the more complex diagnostic types require psychotherapy (1). Therefore, a psychometrically-sound measure of the child’s level of anxiety is required in order to use an appropriate anxiety-reducing intervention with a given child.

There are a number of methods employed to assess childhood distress in general, and pain, anxiety and/or fear in particular including observer-rated, self-report, parental-report and physiological measurements (2-4).

The idea is that different measurement techniques may illuminate different aspects of the stressful experience. Specifically, observational scales can quantify children’s overt behavioral manifestations of affective and sensory distress; parental- and staff-ratings can highlight adults’ perceptions of children’s distress; and self-reports can tap children’s perceptions of their own distress. Despite the value of self-reports, children’s report is limited by their developmental level, response bias, and situational demands that must be controlled in order to obtain adequate assessments (5). Most of the self-report measures available to date are downward extensions of adult measures of anxiety and are based on the assumption that anxiety in children closely resembles the presenting features of anxiety in adults (6). Thus, there seems to be an assumption with most of these measures that children experience and report anxiety in the same way as adults. Other limitations of self-report measures include young children’s inability to perform well on self-report approaches, and more time is required to complete the questionnaire (5).

Behavioral/observational methods are the primary approach for assessing anxiety in young children. An important issue is whether the measures capture behavioral alarms that represent anxiety. Another issue is the lack of attention to developmental differences in behavioral manifestations for infants, children, and adolescents. Findings from studies demonstrate that age or developmental level affects the overt manifestation of some behaviors like vocalizations and large motor movements. Previous studies concluded that younger children undergoing invasive procedures exhibit more crying behavior than older children (7).

Physiological approaches generally rely on interpretation of changes in several physiological parameters include hormones and metabolites, endorphins, vital signs (heart/pulse rate, respiration rate, and blood pressure), and diaphoresis as indicators of pain and anxiety. However, there is insufficient evidence to conclude that physiological responses correlate directly with pain and anxiety experience. Although there have been only a few studies examining the physiological parameters of anxious mood in children, most researchers have found that the physiological responses by normal children to fear-producing or threatening situations are similar to those found in adults (8). The results of the studies by Tiwari et al. confirmed the physiological changes in the body as a result of the anxiety and stress during dental treatment (9). Although measurement of these physiological variables is possible in children, there are several difficulties including the paucity of norm measurements, the inconvenience and expense of the necessary sophisticated equipment, and the necessity of child’s cooperation in some procedures (4,8).

To overcome these shortcomings, several investigators have used psycho-physiological measurements such as galvanic skin response (GSR) to quantitate levels of anxiety in patients (10). It has been showed that GSR is an extremely accurate objective method and has been used in various studies to measure dental anxiety (11). A psycho-galvanometer measures the conductance of the skin of passage of a very small electric current (12). Electrical changes are induced by minute amount of fluid from epidermal sweat glands released secondary to anxiety. Sweat on the skin provides a low-resistance pathway for electrical current, which is then recorded (11). It has been known for decades that the magnitude of this electrical conductance is affected not only by the subject’s general mood but also by immediate emotional reactions which are used in psychophysiology experiments to infer emotional state and cortical arousal in response to stressful situations (12,13). Although skin conductance has mainly been used to evaluate the effect of chronic and acute stressful stimuli, it has also been used in the study of social interaction (12). The results of the two studies in adult patients showed that the skin conductance levels were significantly different in dental patients with stress-inducing stimuli (10,14). Therefore, this study aimed at evaluating the correlation between galvanic skin response (GSR) and two well-established anxiety assessment tools namely the Modified Child Dental Anxiety Scale (MCDAS), and the Clinical Anxiety Rating Scale (CARS) to provide evidence of the validity and reliability of the GSR in pediatric dental patients.

## Material and Methods

The protocol for this study was independently reviewed and approved by the Institutional Review Board at the university. The participants included 5 to 7-year-old children enrolled in the Department of Pediatric Dentistry, Tabriz University of Medical Sciences, during the period from January to June 2016. They were referred for comprehensive assessments as well as routine dental treatments. Once admitted, these children were examined by a post-graduate student under the supervision of a pediatric dentist. A comprehensive medical and dental history was taken and a treatment plan was established for each patient (15,16).

Following criteria were considered for inclusion in the study: Complete physical and mental health without any confounding medical history. No history of unpleasant experiences in medical settings. No history of post-traumatic stress disorders or specific phobia related to dental settings. No previous experience of intra-oral injections. Existence of carious primary teeth which needed restorative treatment.

According to the pilot study conducted on 20 patients and considering α equal to 0.05 and prevalence of dental anxiety equal to 20% with maximum marginal error of 8% (3,17), and assuming 80% sensitivity between the GSR and CARS, 126 samples were needed for this study which were increased to 151 to improve the validity and power of study. The pilot cases were not included in the main study. After preliminary selection of 200 patients who matched the inclusion criteria of the study, a total of 151 patients were randomly included in the study. Study procedure was explained to the parents and an informed written consent was taken. The study procedure was approved by the research and ethics committees of the Tabriz University of Medical Sciences. All subjects received a mandibular primary molar restoration after local anesthesia in the treatment session.

-Assessment instruments

Three measurements were taken: the Modified Child Dental Anxiety Scale (MCDAS) as a self-report scale (18), the Clinical Anxiety Rating Scale (CARS) as a behavioral scale (19), and the Galvanic Skin Response (GSR) for physiological evaluation of dental anxiety.

**Figure 1:**
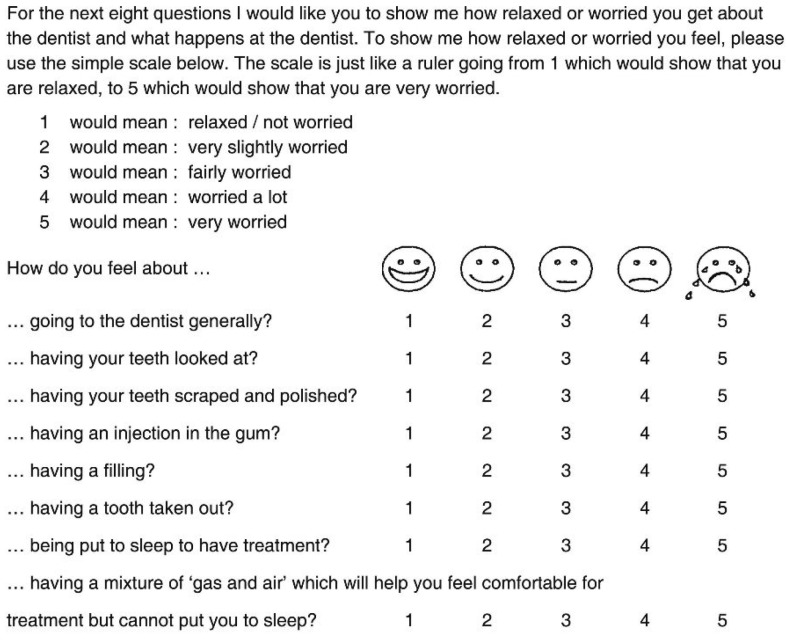
Faces version of the Modified Child Dental Anxiety Scale (MCDAS).

MCDAS is a measure of dental anxiety in children (Fig. 1). It has an 8-question format, with a numeric rating scale ranging from 1 (relaxed/not worried) to 5 (very worried). Thus, the total score may range from 8 to 40. Sufficient evidence has been provided to justify the test reliability and validity (18). Children’s parents were present during the measurement. However, the objective of the study was described to the parents and they were instructed not to influence the children to respond one way or the other.

CARS is a six-point rating scale and the scores range from 0 to 5 ( Table 1) (19). The raters received adequate training prior to this study to become familiar with the rating scales used.

**Table 1:**
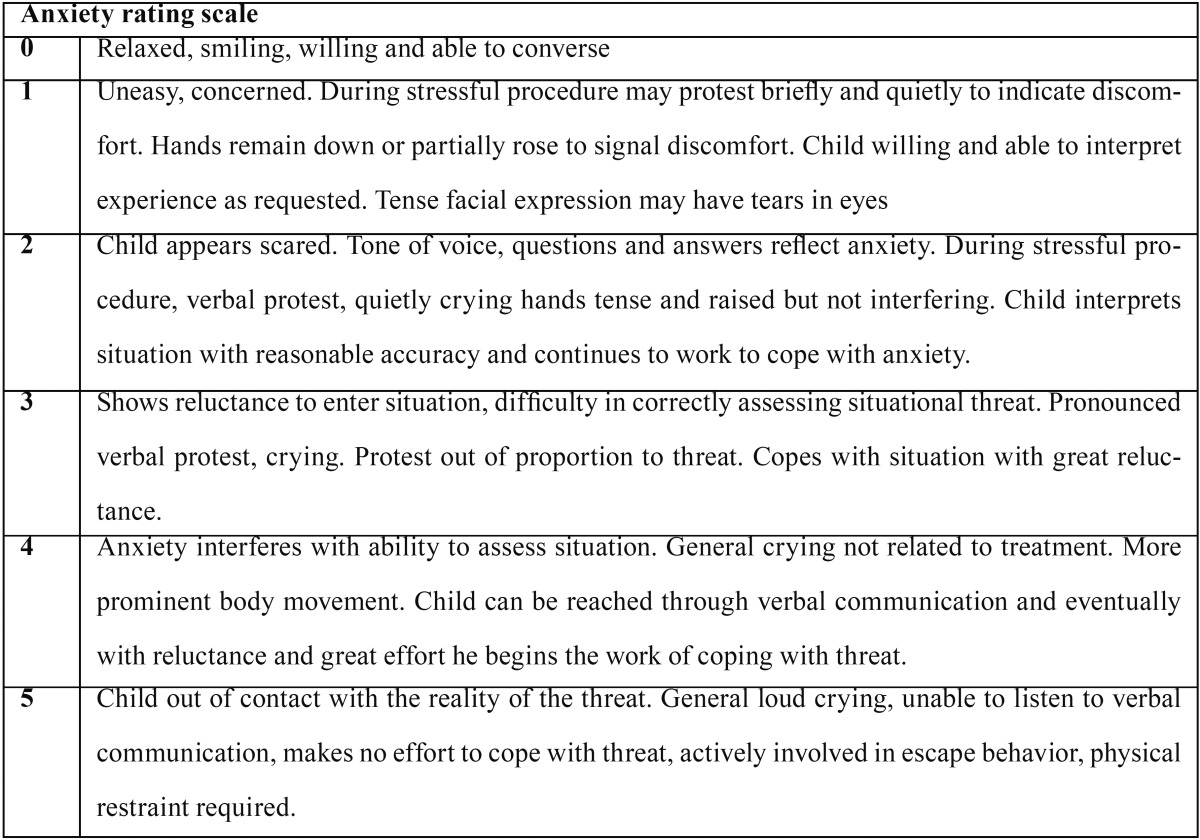
Clinical anxiety rating scale.

The GSR was measured by attaching two cupper electrodes to the distal phalanges of the index and middle finger of the subject’s left hand. Patients washed their hands with water and soap and dried them thoroughly before treatment. The patient’s arm rested on the armrest of the dental unit to support the arm and hand to avoid signal artifacts, which may arise from movement of the hand to which the electrodes are attached. The current was amplified and measured by a digital readout V/ohm meter (GSR Biofeedback Monitor/Meter, UK).

The dental treatment was rendered for all subjects by the same pediatric dentist. All patients were seen between 8 in the morning and 12 noon to adjust for diurnal fluctuations in eccrine sweat-gland activity (14). GSR readings were recorded at the beginning and at the end of the treatment session. MCDAS and CARS were also rated at two points by a pediatric dentist and a postgraduate student, separately and independently. The inter-rater agreement was excellent (Kappa=0.81).

For evaluation of test-retest reliability, 20 subjects who needed an almost similar treatment, e.g. a restoration on the opposite side, were randomly selected for second observation. The average retest interval between the initial and second treatment was 14 days with a standard deviation of 2 days.

-Data analysis 

Descriptive statistics were used for reporting the data. The data were evaluated by the Kolmogorov-Smirnov test and Q-Q plot to check the normal distribution of data. Levene’s test was used to assess the equality of variances (20,21). Spearman’s correlations coefficient, Wilcoxon signed-rank and paired samples t tests were used for evaluation of relationships between quantitative variables. The receiver operating characteristic (ROC) curve was used to determine the sensitivity and specificity of GSR according to the MCDAS and CARS as the standard scales (3). Statistical analysis was performed with SPSS 16 software for Windows (SPSS, Chicago, USA). Statistical significance was considered when *P*<0.05.

## Results

151 children (58 girls and 93 boys) with the mean age of 5.69 ± 0.76 were included in the study. The mean age for girls was 5.58 ± 0.72 and the mean age for boys was 5.76 ± 0.78. This age difference was not statistically significant. Test-retest data from 20 subjects (8 boys and 12 girls) across a two week period showed a strong significant agreement (*p*<0.001).

The mean GSR reading was 67.93 ± 13.76 at the beginning and 61.43 ± 14.48 at the end of the treatment. The mean score for MCDAS was 21.4 ± 9.52 and 19.55 ± 6.79, respectively. These values for CARS were 2.86 ± 0.67 and 1.54 ± 0.87, respectively ( Table 2). A statistically significant decrease in GSR readings was detected at the end of the session (*p*= 0.001). Similarly, a significant decrease in the MCDAS score was found at the end of the treatment (*p*= 0.001). CARS also showed a reduction between the two assessments, but it was not statistically significant (*p*= 0.36).  Table 3 shows that all three measures revealed no significant differences in dental anxiety by gender.

**Table 2:**
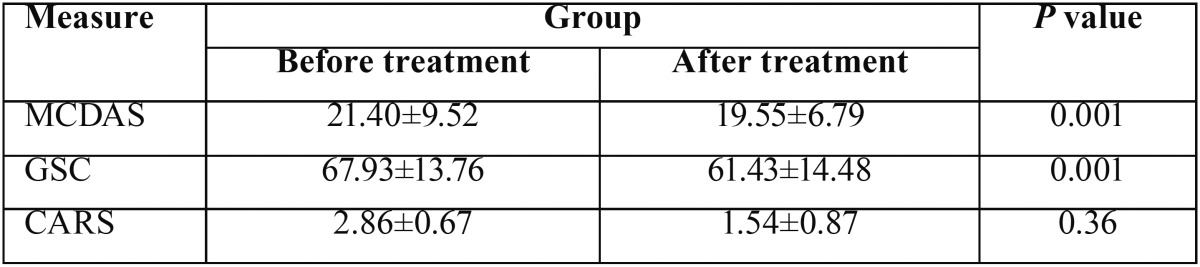
Mean scores on the Dental Anxiety Scales and the skin-conductance levels at the beginning and end of the treatment session.

**Table 3:**
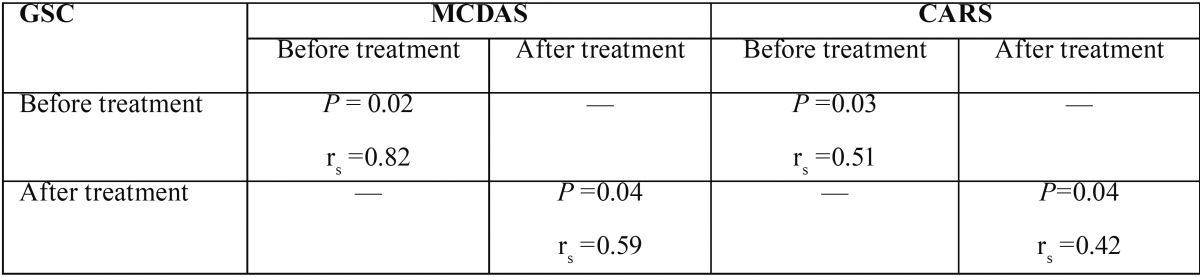
Spearman’s correlations of GSC readings with MCDAS and CARS scores.

Spearman’s test showed that there was a statistically significant correlation between the mean GSR reading and mean MCDAS score at the beginning (rs = 0.82, *p*= 0.02) and at the end of the treatment (rs = 0.59, *p*= 0.04). In the same line, there was a statistically significant correlation between the mean GSR reading and mean CARS score at the beginning (rs = 0.51, *p*= 0.03) and at the end of the treatment (rs = 0.42, *p*= 0.04) ( Table 4). Spearman’s test also revealed a statistically significant strong correlation between the total mean GSR readings and the total mean MCDAS (rs = 0.62, *p*= 0.02) and CARS scores (rs = 0.44, *P*= 0.03). The correlation between MCDAS and CARS was also significant (rs = 0.9, *P*< 0.001). The sensitivity and specificity of GSR was respectively 74% and 83% (*p*<0.001) based on the MCDAS; and 78% and 89% (*p*<0.001) based on the CARS tests.

**Table 4:**
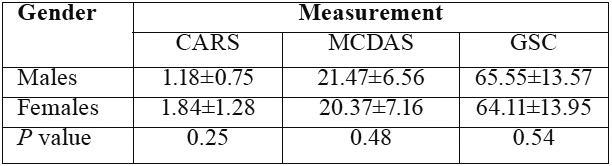
Mean dental anxiety scores according to three measures evaluated.

## Discussion

The present study aimed to determine the reliability and validity of GSR as a measure for anxiety assessment in children. The GSR uses psycho-physiological measurements to quantitate levels of anxiety in patients (10). The first step of validation was to compare GSR with the fully recognized scales of anxiety i.e. MCDAS and CARS, which have been shown to be valid measures (18,19).

In the present study, pair comparisons of GSR vs. MCDAS and GSR vs. CARS have revealed a statistically significant correlation. This level of correlation between the measures in the present study confirms the validity of GSR to detect anxiety levels in children during dental treatment.

The results of the present study showed that the mean GSR reading decreased significantly during treatment session. Similar trends were seen with MCDAS and CARS. In the same line, Venham *et al.* concluded that cooperative behavior increased at the second measurement (22). An explanation for this phenomenon is that anticipation of an unknown or unpleasant event is an established cause of anxiety. Participants would have been more familiar with treatment procedure and environment at the second administration (23).

An important property to validate in a scale is the responsiveness to change (3). Sensitivity of the GSR to detect changes in anxiety between the beginning and the end of the treatment session in this study provides further confirmation for its validity.

Despite inconsistent findings in the previous studies regarding the issue in adult subjects, our preliminary results clearly indicate that GSR is a valid and reliable measure of children’s anxiety in the dental setting. In accordance with our findings, several studies have shown a relationship between self-reported measures of dental anxiety and skin conductance in adults (10,14,24). It has been shown that in the highly-anxious patients the correlations among scores are stronger. In contrast, other studies in adult patients did not show a relationship between self-reported measures of dental anxiety and GSR (level and nonspecific fluctuations) or the reciprocal measures of conductance, that is, skin resistance (24-27).

The results of the present study also revealed no gender differences for overall anxiety scores of the three scales used. However, there is an inconsistency in the literature regarding gender differences. Some studies have shown a clear distinction between males and females (girls indicating raised dental anxiety over boys) (28,29) while other work showed no differences (30,31). However, gender-related effects in GSR have not been established (32).

GSR has a number of advantages which may encourage assessment of anxiety using this assessment tool. First, the GSR is quick and easy to administer in the dental setting. It takes a very short time to administer and gives an immediate ‘state’ feedback to the clinician in the dental environment which allows the clinician to design appropriate treatment plans including accurate behavior management strategy for their child patient. Second, it does not need scheduled time and can be administered during treatment procedure. Third, the GSR can be employed with very young children. It has been noted that a stalemate situation arises with very young children where their lack of cognitive ability means they cannot complete questionnaires (33). In these patients, indirect psycho-physiological measures are the only real alternative. Whilst the self-report measures and assessment of behavioral responses to anxiety produce valuable information; these responses are also affected by many factors other than anxiety (33,34). Measurements of psycho-physiological responses in children during dental procedures have demonstrated a general pattern of sympathetic arousal with increased secretion of catecholamines, increased heart rate, and decreased galvanic skin resistance (35). Physiological indicators of anxiety may be relatively less susceptible to unreliability due to unconsciously and/or consciously mediated attempts to deny the effects of stressful situations than the signs on the verbal-cognitive level. GSR is a comparatively robust physiological measure that can be measured relatively inexpensively, easily, non-invasively, and unobtrusively which is unaffected by cardio-respiratory status (33,34).

Although the GSR evaluates child anxiety using psycho-physiological response, such conclusions should be weighed carefully, considering the fact that children’s behavior and/or anxiety can have different aspects with various levels of alarm. In addition, 5 to 7-year-old Children were examined in the present study and it is plausible that assessment of children’s GSR during other developmental periods may yield a different pattern of findings. Moreover, participants at the first administration are not familiar with the instrument and it may results in stress. The limitation for this instrument is diurnal fluctuations in eccrine sweat-gland activity. Therefore, the time of assessment can influence results. Temperature and humidity can also influence skin conductance (36). Thus, the extrapolation of the results of the present study to a broader sense and generalization of the findings necessitates further investigation.

## Conclusions

In conclusion, the findings of present study proved the validity and reliability of the galvanic skin response in children in the dental setting. We have, however, argued that no instrument met all of the criteria identified as necessary for measurement of child’s dental anxiety. The GSR may particularly be an effective assessment tool when used in conjunction with other behavioral and/or self-report scales, which increases the likelihood of capturing children’s dental anxiety.
